# Hypoxia-Inducible Factors as Key Players in the Pathogenesis of Non-alcoholic Fatty Liver Disease and Non-alcoholic Steatohepatitis

**DOI:** 10.3389/fmed.2021.753268

**Published:** 2021-10-06

**Authors:** Lorenz M. W. Holzner, Andrew J. Murray

**Affiliations:** Department of Physiology, Development and Neuroscience, University of Cambridge, Cambridge, United Kingdom

**Keywords:** non-alcoholic fatty liver disease (NAFLD), non-alcoholic steatohepatitis (NASH), hypoxia-inducible factor (HIF), chronic intermittent hypoxia, obstructive sleep apnea, fibrosis, metabolism, inflammation

## Abstract

Non-alcoholic fatty liver disease (NAFLD) and its more severe form non-alcoholic steatohepatitis (NASH) are a major public health concern with high and increasing global prevalence, and a significant disease burden owing to its progression to more severe forms of liver disease and the associated risk of cardiovascular disease. Treatment options, however, remain scarce, and a better understanding of the pathological and physiological processes involved could enable the development of new therapeutic strategies. One process implicated in the pathology of NAFLD and NASH is cellular oxygen sensing, coordinated largely by the hypoxia-inducible factor (HIF) family of transcription factors. Activation of HIFs has been demonstrated in patients and mouse models of NAFLD and NASH and studies of activation and inhibition of HIFs using pharmacological and genetic tools point toward important roles for these transcription factors in modulating central aspects of the disease. HIFs appear to act in several cell types in the liver to worsen steatosis, inflammation, and fibrosis, but may nevertheless improve insulin sensitivity. Moreover, in liver and other tissues, HIF activation alters mitochondrial respiratory function and metabolism, having an impact on energetic and redox homeostasis. This article aims to provide an overview of current understanding of the roles of HIFs in NAFLD, highlighting areas where further research is needed.

## Introduction

Non-alcoholic fatty liver disease (NAFLD) is a progressive, widespread form of chronic liver disease with a large global burden. Worldwide, around 25% of the population have NAFLD and its prevalence is increasing (1). NAFLD initially presents as relatively benign fatty liver but worsens with time, leading to fibrosis and the inflammatory, more severe non-alcoholic steatohepatitis (NASH). Eventually, even cirrhosis or hepatocellular carcinoma can occur (2). It is also an important independent risk factor for cardiovascular disease (1). Despite this, specific treatment options for NAFLD are lacking. In order to develop such specific treatments, a better understanding of disease mechanisms and the (patho-)physiological signalling systems involved in NAFLD progression are needed.

**Figure 1:**
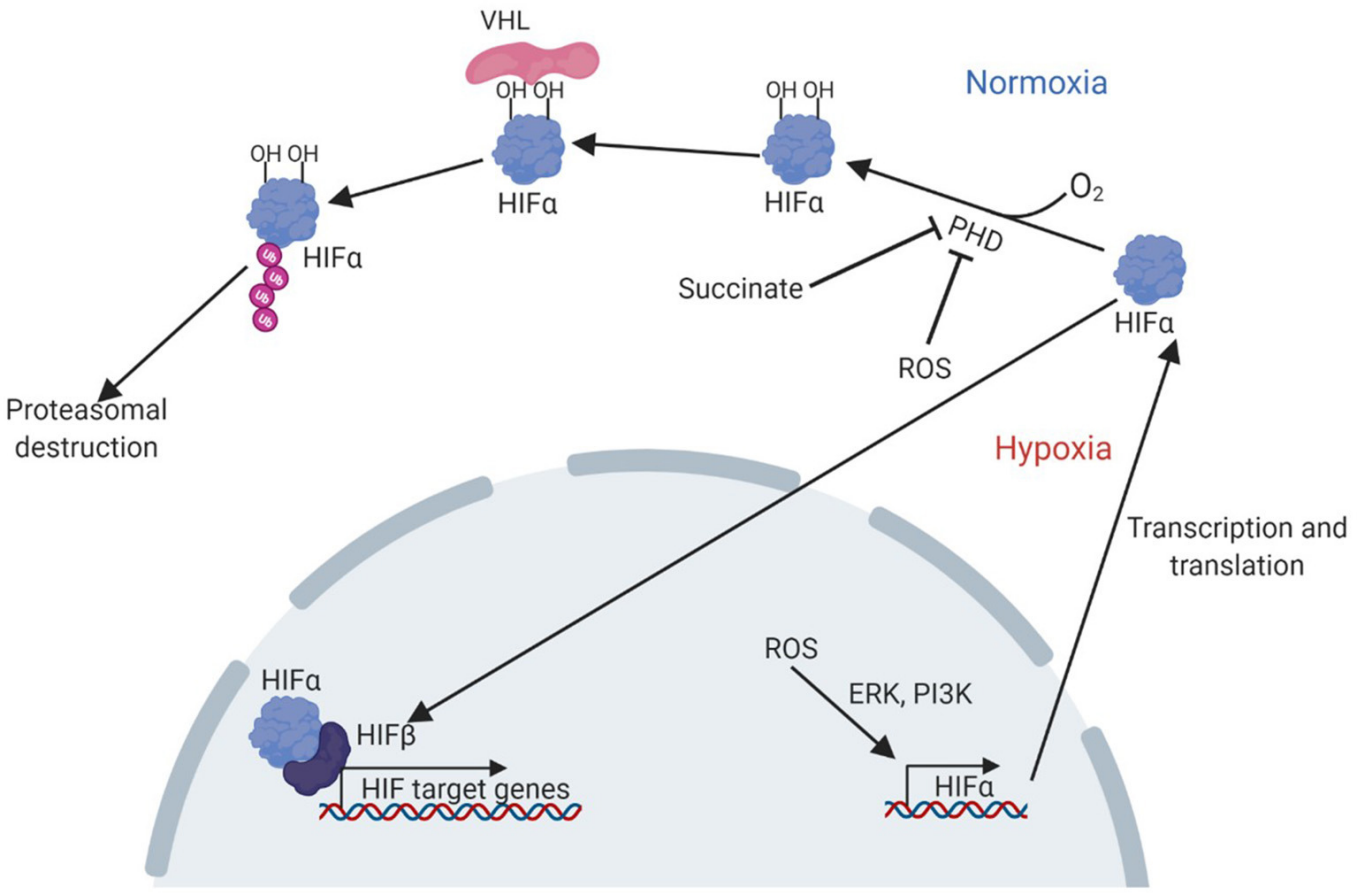
Pathway of HIF activation in hypoxia. Adapted from Lee et al. (7). Under normoxic conditions, PHD enzymes hydroxylate proline residues in the HIFα subunit, in an oxygen-dependent manner. The hydroxylated residues are bound by VHL, which ubiquitinates HIFα allowing recognition and destruction of HIFα by the proteasome. Under hypoxic conditions, hydroxylation cannot occur and HIFα can instead translocate to the nucleus, bind HIFβ and other cofactors to activate transcription of target genes. HIF accumulation can also result from PHD inhibition by succinate or ROS, or by increased transcription and translation due to a ROS induced, ERK and PI3K mediated pathway. ERK, extracellular-signal related kinase; HIF, hypoxia-inducible factor; PHD, prolyl hydroxylase domain proteins; PI3K, phosphoinositide 3-kinase; VHL, Von Hippel Lindau protein.

The hypoxia-signalling system has been implicated in the pathogenesis of NAFLD (3). Central to cellular oxygen-sensing is the hypoxia-inducible factor (HIF) family of transcription factors which regulate the expression of genes underpinning the cellular and systemic response to hypoxia. HIFs are heterodimers, made up of an alpha subunit (of which three are currently known: HIF1α, HIF2α, and HIF3α), and a beta subunit (HIF1β). Current understanding of the regulation and function of HIF1α and HIF2α, is much greater than that of HIF3α, which remains under-investigated (4). The 2019 Nobel Prize in Physiology or Medicine was awarded to William Kaelin Jr., Peter J. Ratcliffe, and Gregg L. Semenza for their work in revealing how HIFs sense oxygen levels and coordinate the cellular response to hypoxia. The sensing mechanism, which has been reviewed elsewhere (5), involves targeted destruction of HIFα subunits in the presence of oxygen (Figure 1). Under normoxic conditions, HIF-prolyl hydroxylase domain proteins (PHD1-3) hydroxylate proline residues in cytoplasmic HIFα subunits in an oxygen-dependent manner. This allows recognition by the E3 ubiquitin ligase von-Hippel Lindau protein (VHL), leading to ubiquitination of HIFα and subsequent proteasomal degradation. PHD-mediated hydroxylation does not occur in hypoxia, allowing HIFα stabilisation, translocation to the nucleus and dimerization with HIF1β. Activated HIFs bind to hypoxia response elements in the promoters of target genes, leading to the transcription of genes required for adaptation to hypoxia, such as *Vegfa*, encoding vascular endothelial growth factor, and genes encoding many glycolytic enzymes (6). Owing to their roles in the regulation of diverse processes such as metabolism and angiogenesis, there is great potential for the involvement of HIFs in multiple key aspects of NAFLD, and accumulation of HIFs has been demonstrated to occur in the livers of patients with NAFLD (3). This makes HIF signalling a promising therapeutic target for this disease, especially since pharmacological modulators of the HIF pathway already exist (7–9).

### Potential Mechanisms of HIF Activation in NAFLD

The canonical driver of HIF activation is tissue hypoxia. Hypoxia in the liver has been shown to occur in mice fed a high fat diet (HFD) for 8 weeks, though it remains unclear how this local hypoxia develops (10). The liver displays a steep oxygen gradient, with higher partial pressures of oxygen in the periportal regions, but lower oxygenation in perivenous regions (11). In NAFLD, this gradient could become dysregulated, leading to hepatic hypoxia, and this has been observed using pimonidazole staining in mice fed a HFD (12). Pimonidazole is a small molecule that reacts with thiol groups in proteins and peptides specifically under hypoxic conditions allowing for the detection of hypoxia using immunohistochemical techniques (13). Dysregulation of the oxygen gradient in the liver could result from increased size of hepatocytes (which increases the diffusion distance for oxygen), e.g., due to steatosis, or from increased oxygen consumption, which may occur in early stages of NAFLD development as appears to be the case in HFD fed rats (14, 15). This increase in oxygen consumption may be a result of increased fat oxidation to avoid lipid accumulation in a state of high fat intake. In addition to hypoxia, HIF stabilisation also occurs in response to reactive oxygen species (ROS) production (16), which is commonly seen in animals fed a HFD (17), and can be caused by cholesterol accumulation (18). ROS production could also result from reduced levels of the sirtuin SIRT4, which have been observed in patients with NAFLD (19). HIF activation can also result from succinate accumulation (20). SIRT1 has also been shown to be an important component of HIF activation (21). However, it should be noted that SIRT1 is generally downregulated in patients with NAFLD (22), and it is therefore unclear whether this mechanism is involved in regulation of HIFs in this context. While localised hypoxia has been demonstrated in steatotic mouse livers (10), it remains unclear whether this is driven by increased diffusion distance, increased oxygen consumption, or a combination of both. Further, other mechanisms of HIF activation, such as ROS production and importantly, chronic intermittent hypoxia (CIH), remain under-investigated in this context. CIH occurs in humans with obstructive sleep apnoea (OSA), which causes nocturnal bouts of low blood oxygen caused by breathing difficulties (23). It is common in patients with obesity (24), and has been linked to NAFLD severity (25), but it remains unclear to what extent it is required for HIF activation in patients with NAFLD, and whether HIF mediated pathophysiological mechanisms differ between patients of NAFLD with and those without OSA. It should be noted that while rodents do not spontaneously develop OSA (meaning CIH does not occur in rodent models of NAFLD), HIF accumulation has been demonstrated in the livers of rodent models of NAFLD. This supports the view that CIH is not necessarily a requirement for HIF activation in NAFLD. The uncertainty around the mechanism driving HIF activation in NAFLD is of note, as mechanistic into this very common disease remains lacking (26), making it crucial to address such gaps in our understanding of the pathology of NAFLD.

### Metabolic Roles of HIFs in NAFLD

Regulation of cellular metabolism is a major canonical function of HIFs. In order to maintain energy charge in hypoxia, HIFs increase the expression of genes encoding glycolytic enzymes such as lactate dehydrogenase (27), while repressing the expression of genes involved in oxidative metabolism, particularly fatty acid oxidation (FAO) (28). This serves to decrease oxygen requirements for ATP production, and protects against cellular damage in short-term hypoxia. However, chronic activation of HIFs in patients and models of fatty liver disease (3) may inhibit FAO to such an extent that it leads to or worsens hepatic lipid accumulation. HIF activation also appears to worsen steatosis by increasing the expression of genes required for lipogenesis, and the uptake and storage of lipids (9). Under normal circumstances, this may be an adaptive response to acute hypoxia, acting to store energy sources that cannot be utilised due to the general limitation on oxidative metabolism, and to package potentially toxic fatty acids as less harmful triglycerides. Overall, however, the resulting lipid accumulation appears to represent a harmful role for HIFs in steatotic liver diseases, such as NAFLD. It may also explain part of the association between severe OSA severity and incidence of NAFLD (29). Evidence of an insulin-sensitising role of HIFs in metabolic disease (30) complicates the overall effect of HIF activation in fatty liver disease, which is typically associated with insulin resistance (31).

**Figure 2:**
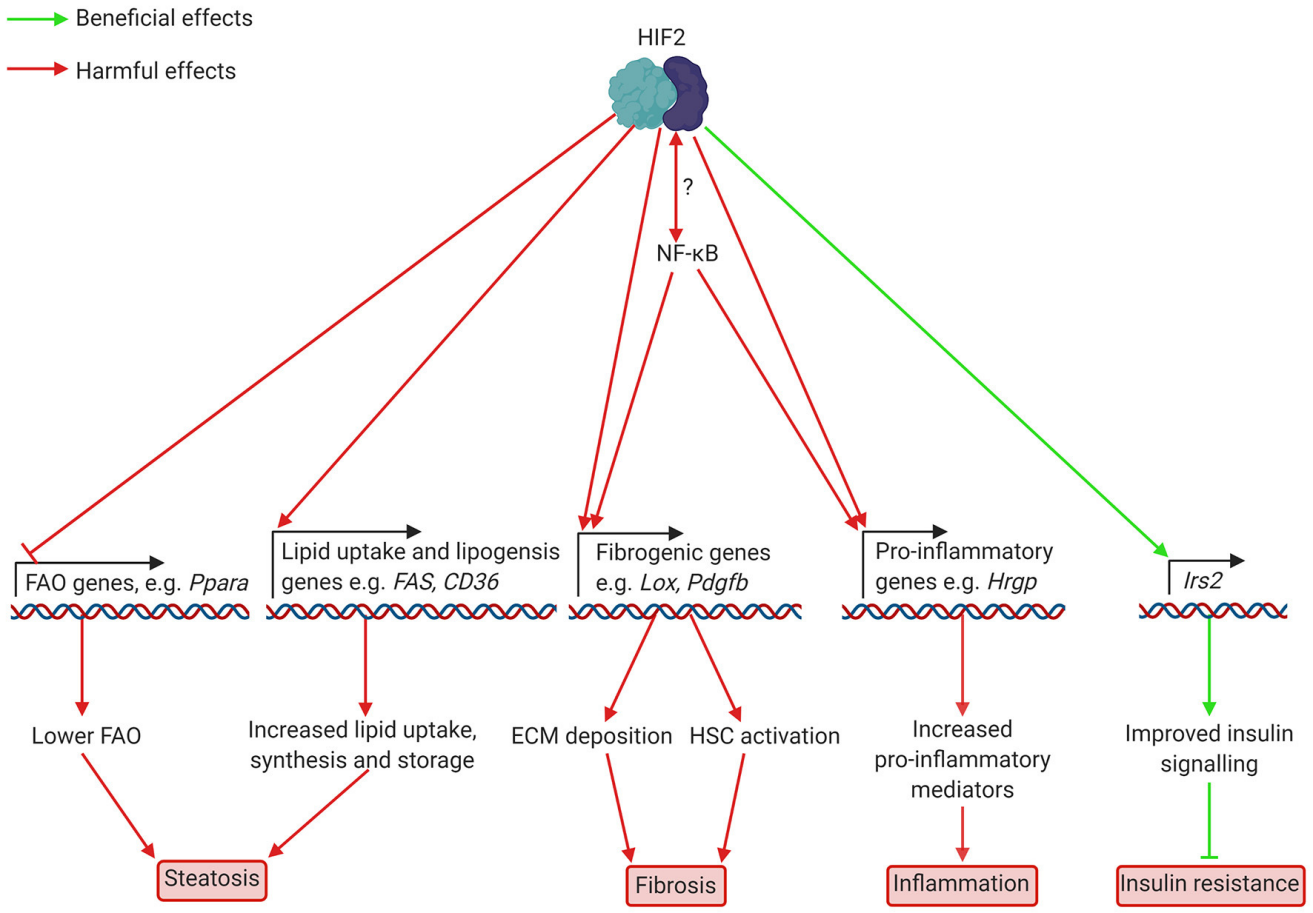
Putatively beneficial and harmful effects of HIF2 activation in hepatocytes in NAFLD and NASH. HIF2 activation leads to lower expression of FAO genes, including *Ppara*, which encodes PPARα. This decreases FAO, leading to increased lipid accumulation. Higher levels of fibrogenic mediators such as LOX and potentially PAI-1, which are involved in ECM deposition, also occur as a result of HIF2 activation. Increased production of HSC activators may also occur but this has not yet been demonstrated in NAFLD/NASH. HIF2 mediated upregulation of the pro-inflammatory cytokine HRGP worsens inflammation. Interplay between HIF2 and NF-κB appears likely, but details of this interaction are unknown. Finally, increased transcription of the insulin signalling component Irs2 appears to improve insulin signalling to prevent insulin resistance. ECM, extracellular matrix; FAO, fatty acid oxidation; HIF, hypoxia-inducible factor; Hrgp, histidine rich glycoprotein; HSC, hepatic stellate cell; Irs2, insulin receptor substrate 2; Lox, lysyl oxidase; NF-κB, nuclear factor kappa-light-chain-enhancer of activated B cells; Ppara, peroxisome proliferator-activated receptor α; Fas, fatty acid synthase; CD36, cluster of differentiation 36; Pdgfb, platelet derived growth factor b.

Considerable evidence points toward HIF-mediated downregulation of FAO in hepatic steatosis. In particular, HIF2α activation, which occurs in the livers of patients with NAFLD as well as in mouse models (3), appears to worsen lipid accumulation (see Figure 2). Early studies in *Vhl*-deficient mice, showed that HIF2α, but not HIF1α, is responsible for the suppression of FAO in these mice (32–34). *Vhl-*deficient mice had lower expression of peroxisome proliferator-activated receptor α (PPARα)-target genes, such as carnitine-palmitoyl transferase 1 (*Cpt1*) and acyl CoA oxidase (*Acox*), lowering fatty acid-supported oxidative phosphorylation (33). PPARs are a family of transcription factors activated by unsaturated fatty acids, amongst other ligands. They play a key role in the control of fatty acid metabolism, and PPARα in particular is a major regulator of FAO in the liver (35). The reduced expression of PPARα target genes in *Vhl*-deficient mice was prevented by deletion of *Epas1* (*endothelial PAS domain containing protein 1*, the gene encoding HIF2α) but not *Hif1a* deletion (32). Similarly, primary hepatocytes from *Vhl*-deficient mice showed increased lipid accumulation alongside low expression of PPARα target genes (36). Indeed, HIF2α binds the PPARα promoter to repress its expression in HEK293 cells (28). *Hif2a* deletion or knockdown using siRNA prevents hypoxia-associated lipid accumulation in the human hepatocellular carcinoma HepG2 cell line (37, 38). Hypoxia appears to cause lipid accumulation in these cells by stabilising HIF2α, thereby lowering expression of FAO genes such as *Cpt1* and PPARγ coactivator α (*Pgc1*α) (38). Expression of these genes was normalised by *Hif2a* deletion, leading to decreased lipid accumulation. These studies demonstrate a potential role for HIF2α activation in decreasing the capacity for FAO in the liver, which could worsen steatosis in the context of NAFLD, when dietary fat intake is typically high.

While it is clear that HIF, and in particular HIF2α, activation can limit FAO in the liver to worsen steatosis, the studies outlined above did not investigate whether this occurs in NAFLD. Studies in *in vitro* systems and animal models of NAFLD suggest that this is indeed the case. Mice exposed to a HFD to induce hepatosteatosis showed decreased lipid accumulation when treated with a HIF2α antagonist (9), though FAO was not investigated in this study. In L02 human hepatocytes treated with fatty acids to model NAFLD *in vitro*, hypoxia worsened lipid accumulation, and this was associated with increased HIF2α levels, decreased expression of *Ppara* and transcriptional targets of PPARα such as *Cpt1a* and *Acox*, and lower oxidation of oleate (39). Silencing of *Hif2a* or treatment with a PPARα agonist, normalised expression of FAO genes and oleate oxidation, thereby lowering lipid accumulation, while treatment with a PPARα inhibitor prevented the beneficial effect of HIF2α-silencing. The authors also found that exposure of HFD fed mice to CIH, which models OSA, (see Table 2 for an overview of hypoxia animal and cell culture models) increased lipid accumulation in the liver and decreased the expression of FAO genes including *Ppara, Cpt1a*, and *Acox2*. PPARα agonist treatment reversed the effects of hypoxia on steatosis. Chen et al. (39) did not investigate whether HIF2α activation played a role in lipid accumulation in the absence of a hypoxic stimulus, although other studies have demonstrated that HIF2α accumulation occurs in animal models of NAFLD without added hypoxia (40). Hepatic *Hif1*α deletion in a mouse model of NAFLD (mice fed a choline deficient diet), however, led to lower Lipin1 mediated PPARα/PGC1α pathway activation, which worsened steatosis relative to wild type mice (41), suggesting HIF1α is required to maintain FAO in NAFLD. Further work is required to determine whether HIF2α activation in NAFLD leads to lower FAO in animal models and human patients, especially in the absence of imposed hypoxia, though current evidence suggests that HIF2α activation in NAFLD contributes to hepatic steatosis, and that HIF2α activation can limit fatty acid oxidation, whereas HIF1α appears to be required for FAO in NAFLD.

Increased lipogenesis is an important feature of NAFLD in human patients (42–44). Again, studies support a potential HIF-mediated upregulation of this process in the context of NAFLD, although the current evidence for this role of HIF is conflicting. Studies of animal models of NAFLD suggest that HIF2α activation in this disease context may drive increased lipogenesis, thus worsening lipid accumulation in the liver (see Figure 2). Treatment with the HIF2α specific antagonist PT2399 lowered hepatic steatosis in HFD fed mice (9), and this was associated with decreased expression of lipogenic genes in the liver. In L02 human hepatocytes treated with fatty acids, hypoxia (1% oxygen in hypoxic cell culture incubators) increased the expression of lipogenic genes such as *Fas* and stearoyl CoA dehydrogenase 1 (*Scd1*), and this was normalised by HIF2α silencing (39). Similarly, mice fed a HFD and subjected to CIH showed increased expression of *Fas* and *Scd1* relative to HFD fed mice not exposed to CIH (39). Conversely, oxygen therapy, which prevented HIF2α accumulation, lowered hepatic steatosis in HFD fed mice, and lipid accumulation in primary hepatocytes exposed to fatty acids. This also normalised expression of lipogenic genes in both *in vivo* and *in vitro* models of hepatosteatosis (40). Thus, it appears that HIF2α-activation, resulting from hypoxia, worsens diet induced steatosis by activating lipogenic gene expression. However, it should be noted that genetic HIF2α activation *via Vhl* disruption has been associated with decreased expression of lipogenic genes such as fatty acid synthase (*Fas*) (32), or, in other studies using the same mechanism, with only a temporary increase in lipogenic gene expression 3 days after the *Vhl* disruption (34). These conflicting results may be due to the differing mechanisms of HIF2α activation. In addition to increasing lipogenesis, HIF2α upregulation in NAFLD appears to increase lipid uptake by upregulating the fatty acid transporter Cluster of Differentiation 36 (CD36) (45). CD36 expression correlates with HIF2α levels in patients with NAFLD, and hypoxia induces CD36 expression in mouse AML12 hepatocytes exposed to hypoxia (45). Therefore, there is evidence that HIF2α activation (*via* genetic manipulation or hypoxia) can cause steatosis *via* inhibition of FAO and upregulation of lipid uptake, that liver hypoxia and HIF2α activation occur in NAFLD, and that HIF2α upregulates lipogenesis in diet-induced steatosis, which worsens lipid accumulation and can be prevented by treatment with HIF2α antagonists. However, whether HIF2α also impairs FAO in NAFLD remains unclear.

OSA also induces metabolic changes that may be mediated by HIF signalling. Levels of the CD36 are higher in the livers of patients with OSA than in those of healthy controls, and correlate with severity of OSA (46). CIH, which mimics OSA, induces the expression of lipogenic genes, such as S*cd1*, and CD36 in wild type (46) and *ob/ob* mice (47). Moreover, CIH increased HIF2α, but not HIF1α levels in HFD fed mice, while decreasing the expression of FAO genes such C*pt1a* (39). Thus, it appears likely that HIF signalling decreases FAO and increases lipid uptake and lipogenesis to worsen steatosis in the context of OSA and CIH, though the link between CIH/OSA and HIF signalling has not yet been established.

**Figure 3:**
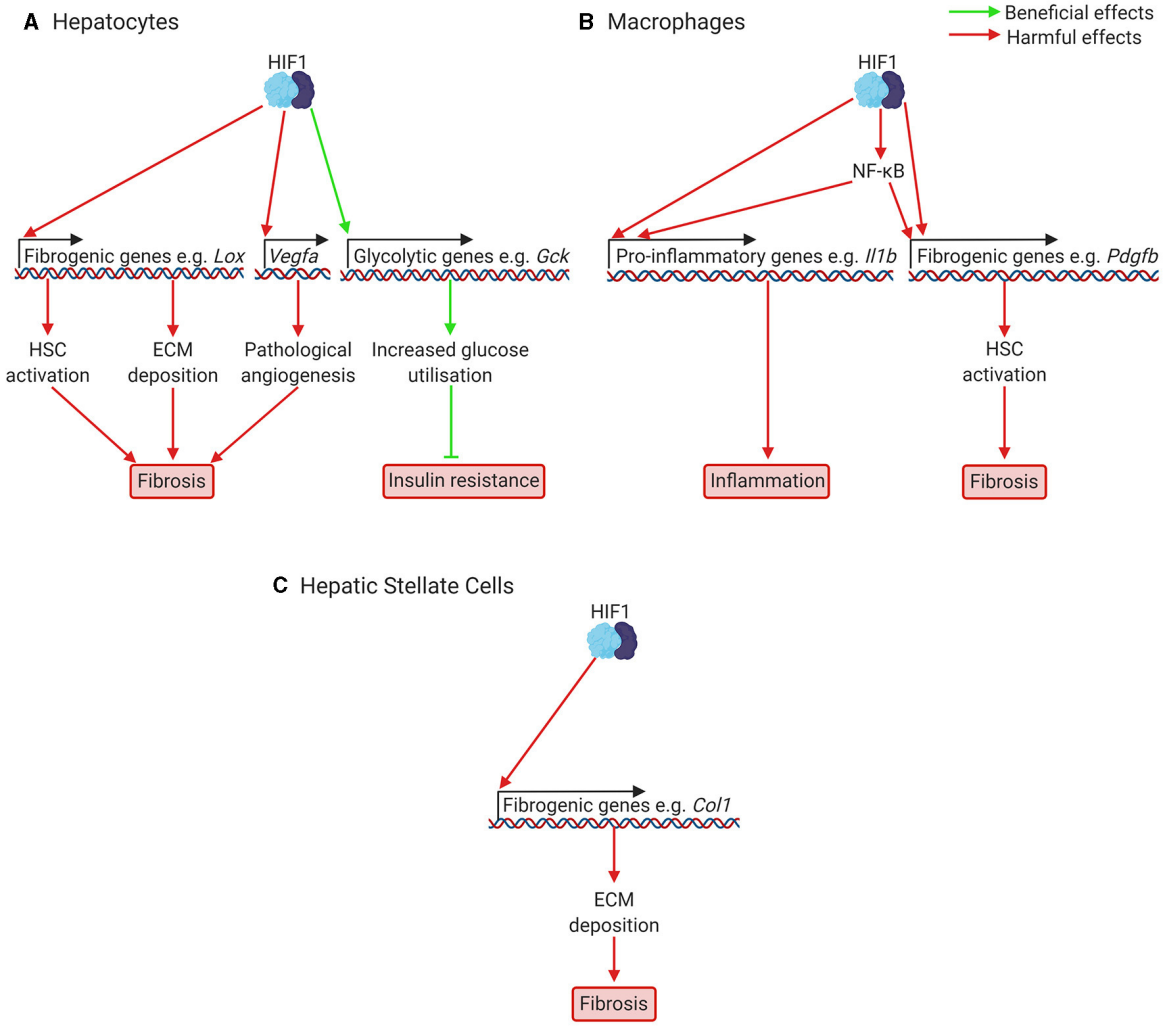
Putatively beneficial and harmful effects of HIF1 activation in NAFLD and NASH. **(A)** In hepatocytes, HIF1 activation leads to increased expression of fibrogenic genes, including genes responsible for HSC activation (e.g., Pdgfb), ECM deposition (e.g., Lox), and pathological angiogenesis (Vegfa). Pathological angiogenesis has only been investigated in fibrosis models, not NAFLD models. HIF activation also increases expression of glycolytic genes such as glucokinase and glucose transporters, which increases glucose consumption and improves glucose handling. This can help prevent insulin resistance. **(B)** In macrophages, including resident macrophages in the liver, Kupffer cells, HIF1 activation increases expression of pro-inflammatory cytokines such as Il1b, leading to inflammation, and of fibrogenic genes, such as the HSC activator Pdgfb, leading to fibrosis. HIF1 also appears to activate NF-κB, a key regulator of fibrosis and inflammation. **(C)** In HSCs, which will be activated at increased levels due to signals from other cells resulting from HIF1 activation, HIF1 activation is also required for transcription of fibrogenic genes, such as Col1, encoding Type 1 Collagen. For many of these effects of HIF1 activation, the precise mechanisms are not yet clear and not all genes mentioned are necessarily under direct HIF1 control. Col1, Type 1 Collagen; Gck, glucokinase; ECM, extracellular matrix; HIF, hypoxia-inducible factor; HSC, hepatic stellate cell; Il1b, interleukin 1b; NF-κB, nuclear factor kappa-light-chain-enhancer of activated B cells; Pdgfb, platelet derived growth factor b; Vegfa, vascular endothelial growth factor A.

The role that HIF activation plays in the context of obesity associated disease is complicated by evidence of a link between HIF and insulin signalling. Both HIF1α and HIF2α activation have been shown to alter insulin sensitivity and glucose handling, most likely in a beneficial manner (see Figures 2, 3). Owing to its role in upregulating glycolytic enzymes, it seems likely that HIF1α could improve glucose handling in obesity and diabetes. Indeed, HIF1α was upregulated in the livers of mice fed a high-fat, high-sucrose diet (30). Hepatocyte-specific deletion of *Hif1a* was associated with worsened glucose handling and insulin sensitivity. This was associated with lower levels of hepatic glucokinase (30). Treatment of HFD fed mice with HIF1α antisense oligonucleotides, however, decreased blood glucose and insulin levels (48). Unlike the hepatocyte-specific deletion employed by Ochiai et al. (30), this not only interfered with *Hif1a* in the liver, but also in adipose tissue, which may explain the opposing results. Shin et al. (48) found increased energy expenditure and lower body weight in *Hif1a* antisense oligonucleotide-treated animals. *Hif1a* antisense oligonucleotide treatment was also associated with lower liver steatosis, increased hepatic *Ppara* expression, and decreased expression of the lipogenic genes *Scd1* and acetyl-CoA carboxylase (48), though again it is unclear whether this was due to *Hif1a* interference in the liver or secondary to effects in other tissues. Overall, it appears that HIF1α activation can have opposing effects on insulin sensitivity, which may be tissue specific. This could explain why OSA severity is associated with worsened insulin resistance in patients with NAFLD (29) while liver-specific deletion of *Hif1a* worsens HFD induced glucose intolerance in mice (30).

HIF2α also appears to be involved in hepatic insulin signalling, *via* direct modulation of components of the insulin-signalling pathway. Liver-specific HIF2α (but not HIF1α) activation led to improved insulin tolerance and glucose handling (49). HIF2α directly upregulates the insulin-signalling pathway component insulin receptor substrate 2 (IRS2) by binding to HREs in its promoter and *Irs2* was required for the HIF2α-mediated effect on insulin sensitivity. Similarly, hepatic deletion of *Phd3*, which specifically upregulated HIF2α, was associated with increased *Irs2* transcription, improving insulin sensitivity (50). Again, this beneficial effect required both *Hif2a* and *Irs2*. *Phd3* deletion was associated with lower expression of gluconeogenic [e.g., phosphoenolpyruvate carboxykinase (*Pepck*)] and lipogenic (e.g., *Fas*) genes. Interestingly, unlike other models of liver specific HIF2α activation, *Phd3* deletion was not associated with worsened steatosis. The authors observed that deletion of *Phd1-3*, which increased HIF2α stabilisation still further, did worsen steatosis, suggesting that lower level HIF2α activation may be predominantly beneficial *via* improved insulin sensitivity, while higher levels of stabilisation, as occurs with *Phd1-3* and *Vhl* deletion (and potentially in long-term NAFLD) has a detrimental effect due to inhibition of FAO, leading to worsened steatosis.

Overall, significant evidence points toward a steatosis-promoting role for chronic HIF2α activation in liver, likely occurring *via* inhibition of FAO and upregulation of lipogenesis, though studies investigating the effect of *Hif2a* deletion in NAFLD on FAO are needed to confirm this. Meanwhile, low levels of HIF2α activation in metabolic diseases appear to have a beneficial effect on insulin sensitivity and glucose handling. Whether HIF1α activation is protective or harmful in the context of metabolic disease and hepatic steatosis remains less clear. There are conflicting results which may be the result of opposing roles in different cell types and tissues, although in hepatocytes specifically, HIF1α activation in obesity appears to improve insulin sensitivity and may be required to maintain FAO and prevent increased lipogenesis.

### HIFs and Fibrosis in NAFLD and NASH

Fibrosis is a key component of NAFLD in its most severe forms (51) and can occur both in patients of non-inflammatory non-alcoholic fatty liver and of inflammatory NASH (52). It is associated with worse outcomes and higher mortality rates in patients with NAFLD (53, 54). HIF-signalling likely contributes to fibrosis in NAFLD as shown by studies of fibrosis in general, and of fibrosis in NAFLD in particular. Liver hypoxia has been demonstrated in animal models of fibrotic and cirrhotic liver disease (see Table 1 for an overview of fibrosis, cirrhosis, and NAFLD models), including in diethylnitrosamine cirrhosis (72), CCl_4_ induced fibrosis (73), bile duct ligation (BDL) (74), and high dietary trans-fat induced NAFLD (56), and increased levels of HIF1α have been found in mouse models (75) and patients with fibrotic liver disease (76). Deletion of *Hif1a* protects against liver fibrosis in mouse models of both fibrotic liver disease, such as mice subjected to BDL (74), and models of NAFLD (56, 77). Similarly, hepatocyte-specific deletion of *Vhl*, which increases both HIF1α and HIF2α signalling led to fibrosis which was normalised by *Hif2a* (but not *Hif1a*) deletion (34), and hepatocyte-specific deletion of *Hif2a* protects against fibrosis in mouse models of NAFLD (3). It therefore appears likely that HIF-signalling contributes to liver fibrosis in NAFLD. HIF-signalling may be involved in fibrosis *via* several mechanisms, including regulation of the expression of fibrogenic mediators in hepatocytes, Kupffer cells (resident macrophages in the liver) and hepatic stellate cells (HSCs) (see Figure 3), and by contributing to aberrant angiogenesis, a process that occurs in parallel with fibrosis and appears to be mechanistically linked to it (78).

**Table 1:** Rodent models for the study of NAFLD.

**Disease**	**Model**	**Aspects of NAFLD/NASH captured**	**References**
NAFLD	High fat diet with varying fat content	Obesity, hepatic steatosis, often insulin resistance, sometimes liver fibrosis, inflammation	(17, 30)
	*ob/ob* mouse	Obesity, steatosis, mild fibrosis	(55)
	*db/db* mouse	Obesity, insulin resistance, steatosis, mild fibrosis	(55)
	High trans-fat diet	Obesity, with steatosis, fibrosis and some inflammation	(56)
NASH	Gubra-Amylin-NASH diet (high fat, high fructose, high cholesterol)	Obesity, severe steatosis, moderate inflammation, moderate fibrosis	(57)
	*ob/ob* mouse with high calorie feeding	Obesity, steatosis, moderate fibrosis, inflammation, moderate fibrosis	(55)
	*db/db* mouse with high calorie feeding	Obesity, insulin resistance	(55)
	Choline-deficient, L-amino acid-defined diet	No obesity, steatohepatitis and fibrosis	(3)
	Methionine/choline deficient diet	No obesity, steatohepatitis and fibrosis	(3, 58)
Cholestatic, fibrotic liver disease	Bile duct ligation	Liver fibrosis	(59)
	Repeated CCl_4_ injection	Liver fibrosis	(60–62)
Cirrhotic liver disease	Diethylnitrosamine injection/feeding	Severe liver injury and cirrhosis, can induce hepatocarcinoma	(63)

**Table 2:** Relevant *in vitro* and *in vivo* models of chronic and chronic intermittent hypoxia.

**System**	**Model**	**Details of model**	**References**
*in vitro*	Cells cultured in hypoxic chambers	Constant hypoxia achieved using high levels of nitrogen. Range oxygen concentrations can be used, 1% most common. Wide range of timeframes.	(64, 65)
	Cells treated with cobalt chloride	Model of HIF activation similar to constant hypoxia. Cellular response sometimes differs from true hypoxia.	(65, 66)
	Cells cultured in hypoxic chambers with cycling oxygen levels	Models CIH *in vitro*. Wide range of oxygen levels at nadir and cycle patterns in use.	(65)
*in vivo*	Rodents in hypoxic chambers	Constant hypoxia achieved using high levels of nitrogen. Range of oxygen concentrations in use.	(67, 68)
	Rodents in hypoxic chambers with cycling oxygen levels	Chronic intermittent hypoxia to model OSA. Oxygen cycles often applied only during sleeping hours of rodents. Wide range of oxygen levels at nadir and cycle patterns in use.	(39, 69)
	Rodents injected with sodium nitrite	Chronic intermittent hypoxemia through methemoglobinemia.	(70, 71)

HIF regulated expression of fibrogenic mediators in hepatocytes has been demonstrated in several relevant *in vitro* and *in vivo* models. Isolated mouse hepatocytes exposed to hypoxia show increased expression of plasminogen activator-inhibitor 1 (PAI-1), and this is partially prevented by *Hif1a* deletion and completely prevented by *Hif1b* deletion, suggesting both HIF1α and HIF2α may be involved (79) (Figures 2, 3). PAI-1 contributes to fibrosis by inhibiting the activities of matrix metalloproteinases, leading to excessive collagen and extracellular matrix (ECM) accumulation (80). Similarly, AML12 mouse hepatocyte cells exposed to hypoxia (81), and HepG2 cells treated with the HIF stabiliser cobalt chloride and free fatty acids (66) show increased expression of genes encoding pro-fibrotic proteins, such as Type 1 Collagen α (COL1A1) and α-smooth muscle actin (α-SMA). In NAFLD models (Table 1), hepatocyte-specific deletion of *Hif1a* protects against collagen deposition and suppresses collagen crosslinking in the media of isolated hepatocytes exposed to hypoxia (56). This is likely to be due to decreased lysyl oxidase (*Lox*) expression, which requires *Hif1a in vitro* (56). *Lox* expression has also been shown by chromatin immunoprecipitation to be under the control of HIF2α (34). In another study of NAFLD models, hepatocyte-specific deletion of *Hif1a* decreased collagen deposition and α smooth muscle actin staining (77). HepG2 cells treated with palmitic acid also showed increased HIF1α levels, and increased Type I Collagen and fibronectin expression, which was prevented by treatment with *Hif1a* siRNA (77). Further, hepatocyte-specific deletion of *Hif2a* protected against fibrosis in choline deficient, amino acid defined diet fed mice, a model of lean NAFLD (3). This was associated with lower levels of *Col1* (Collagen I) and *Acta2* (αSMA) mRNA. Collectively, these studies highlight that one role of HIF activation in liver fibrosis is the direct regulation of fibrogenic genes in hepatocytes and that this likely occurs in NAFLD. However, hepatocytes are not considered major sources of ECM deposition *in vivo*, and so it remains unclear how central this mechanism is to the pathology of NAFLD.

HSCs are the main source of myofibroblasts and therefore fibrosis in liver disease. Myofibroblasts form in the injured liver in response to fibrogenic signals and are the major source of ECM deposition in fibrosis. They are not found in the healthy liver (82). Hypoxia and HIF-signalling appear to play an important role in the activation of HSCs and in regulating the expression of fibrogenic mediators in HSCs. Hypoxia increases the expression of Type I collagen in activated HSCs (72) and HIF-signalling is required for the expression of collagen synthesis genes in isolated HSCs (83) and the production of HSC activators (including platelet derived growth factor (PDGF)-B) in livers in BDL (74), which suggests hypoxia signalling in hepatocytes may play an important role in activating HSCs. Further evidence for this comes from *in vitro* studies; HIF signalling is required for the upregulation of HSC activators in isolated mouse hepatocytes exposed to hypoxia (79), and the conditioned medium of AML12 cells exposed to hypoxia induces α-SMA expression in HSC-T6 cells (81). Similarly, extracellular vesicles isolated from HepG2 cells treated with fatty acids and cobalt chloride to stabilise HIFs induced the expression of fibrotic genes such as Collagen-1 and α-SMA in the human HSC LX2 cell line (66). HSCs are also activated by Kupffer cells and isolated Kupffer cells exposed to hypoxia show increased PDGF-B expression (84). This is normalised by *Hif1b* deletion (84) and myeloid specific deletion of *Hif1a* or *b* protects against fibrosis in BDL (85). Overall, evidence suggests that HIF-signalling is involved in HSC activation by acting directly in HSCs to increase expression of fibrogenic mediators, as well as by increasing the expression of signalling factors that activate HSCs in hepatocytes and Kupffer cells, though this has not been investigated in the context of NAFLD *in vivo* and the relative importance of HIF1 and HIF2 remains unclear.

A further important mechanism linking fibrosis and HIF-signalling is pathological angiogenesis; a common feature of fibrosis and cirrhosis that appears to be closely linked to fibrosis (78). Physiologically, angiogenesis is an important feature of the adaptive response to hypoxia, and is especially vital in liver regeneration after injury, to enable blood supply to re-growing liver regions. It is largely driven by HIF1α-mediated expression of VEGF and treatment with the PHD inhibitor DMOG increases the speed of liver regeneration in rats after portal vein ligation and parenchymal transection, and portal vein ligation alone (86). In pathological or aberrant angiogenesis however, immature neovessels form, which are incapable of resolving localised hypoxia in liver disease, and may lead to chronic HIF activation. Aberrant angiogenesis is likely mediated by increased VEGF expression in fibrosis due to activated HIF signalling (72). Anti-angiogenic treatment with VEGF neutralising antibodies or the VEGF Receptor 2 inhibitor sorafenib can prevent fibrosis in BDL models of liver fibrosis (87, 88), although VEGF may also play a role in fibrosis resolution (88). VEGF expression is increased in hypoxic hepatocytes in a HIF1α-dependent manner (79) and in hypoxic Kupffer cells in a HIF1β-dependent manner (84). VEGF-signalling is highly active in HSCs from areas of active fibrogenesis in patients and animal models, and VEGF stimulates HSC chemotaxis (89). T6-HSCs exposed to hypoxia have reduced levels of VHL, resulting in increased HIF1α and VEGF expression, which is normalised by cyclooxygenase 2 inhibition (90). Thus, chronic HIF-activation may contribute to fibrosis by upregulating VEGF, which contributes to HSC activation and leads to aberrant angiogenesis. However, this has only been investigated in models of fibrotic liver disease, rather than non-alcoholic or metabolic associated fatty liver disease, and further work is required to determine whether pathological angiogenesis is also involved in these conditions.

Hypoxia signalling may also be linked to fibrosis *via* interaction with nuclear factor (NF)-κB signalling. NF-κB is thought to be an important driver of fibrosis and inflammation in NAFLD (91) and inactivation of NF-κB, in particular in Kupffer cells, protects against fibrosis in mice injected with CCl_4_ (60). NF-κB signalling is also activated in HSCs and myofibroblasts in the livers of CCl_4_ and BDL rodent models, and human patients of fibrotic liver disease (92). There is considerable evidence of crosstalk between NF-κB and HIF signalling (93, 94), particularly in immune cells (95). However, the specific link between HIF and NF-κB signalling in the context of NAFLD remains less clear. While it has been demonstrated that both HIF2α and NF-κB accumulate in the livers of patients with NASH and mice exposed to hypoxia (96), it is unclear whether their respective signalling pathways interact and whether modulation of either can affect the other, and thereby improve fibrosis.

A link between OSA and liver fibrosis in NAFLD also appears likely. In patients with OSA and obesity, more severe OSA was associated with worsened fibrosis (29), and circulating levels of LOX (which is regulated by HIFs) are higher in patients with obesity and more severe OSA (64). In mice fed a high trans-fat diet and exposed to CIH (97), and in rats fed a HFD to induce NASH and injected with sodium nitrite to mimic CIH (70) fibrosis worsened. It is not clear what mechanisms contributed to this. In the NASH rat model (70), sodium nitrite injection was associated with increased HIF1α, VEGFA and VEGF receptor 2 levels. Silencing of HIF1α, however, normalised VEGFA and VEGF receptor 2 levels and improved fibrosis, suggesting pathological angiogenesis driven by HIF1α signalling may play a role. VEGF receptor neutralising antibodies attenuated the development of fibrosis in CCl_4_ induced fibrosis, and VEGF stimulated HSC proliferation *in vitro* (98), further supporting a role for pathological angiogenesis. However, the link between CIH and fibrosis may not always be HIF1α mediated, as, while HIF1α deletion improved liver fibrosis and inflammation in trans-fat diet fed mice with or without CIH, it did so without significant interaction with CIH effects (97). Further research is needed to understand the mechanisms involved in the link between CIH/OSA and liver fibrosis.

### HIFs and Inflammation in NAFLD and NASH

Hypoxia is a common feature of chronically-inflamed tissue, and, as highlighted by a number of recent reviews (99–102), HIFs play important roles in inflammation and immunity, including *via* the activation of macrophages and certain types of T cells, and regulation of inflammatory cytokine expression, partly mediated *via* crosstalk with NF-κB signalling (103). Current evidence suggests that both HIF1α and HIF2α play a harmful role in NASH (Figures 2, 3), a more severe form of NAFLD with marked liver inflammation (104). This involves hepatocyte-specific and immune cell-specific roles of HIFs. Hepatocyte-specific normoxic activation of HIF1α and HIF2α *via* deletion of *Vhl* worsens lipid accumulation, fibrosis and inflammation, with global microarray expression analysis showing increased expression of proinflammatory cytokines (34). This pathological phenotype was averted by concomitant deletion of *Hif2a*, but not *Hif1a*, suggesting a greater importance for HIF2α in driving steatohepatitis in hepatocytes. Similarly, in patients with NAFLD, hepatic levels of HIF2α and HIF1α are increased in early, non-inflamed stages of NAFLD, but only HIF2α levels are further increased in the livers of patients with NASH vs. patients with non-inflamed NAFLD (40), though studies in animal models do suggest a possible role for HIF1α as well (97).

In NASH, treatment studies and genetic interference with the HIF pathway point toward HIF activation contributing to inflammation. Treatment with the cardiac glycoside digoxin suppressed HIF1α pathway activation and decreased neutrophil and monocyte infiltration, as well as liver damage, in a mouse model of NASH (105). Further, HIF1α was increased in macrophages from patients and a mouse model of NASH (106). Myeloid specific HIF1α stabilisation worsened steatosis and inflammation, with increased macrophage infiltration in the liver, higher expression of the proinflammatory cytokines macrophage chemoattractant protein 1 (MCP1) and interleukin (IL)-1b in liver macrophages, and higher hepatic levels of *Mcp1* and tumour necrosis factor α (*Tnfa*) mRNA. Palmitic acid treatment also induced HIF1α in macrophages *in vitro*, and silencing of *Hif1a* suppressed the activation of NF-κB (106). HIF2α, which is also increased in patients and mouse models of NASH, appears to influence liver inflammation *via* control of hepatocyte production of the cytokine histidine rich glycoprotein (HRGP) (3). HRGP induces a proinflammatory M1 phenotype in macrophages, and deletion protects against NASH in methionine-choline deficient diet fed mice (107). Choline-deficient, amino acid-defined diet feeding, another model of NASH, increased levels of HRGP and other proinflammatory cytokines (including TNFα) in mouse livers. This was prevented by *Hif2a* deletion, whilst overexpression of *Hif2*α increased HRGP levels in HepG2 cells (3). It therefore appears that both HIF1α and HIF2α contribute to inflammation in NASH, and that this involves HIF-mediated mechanisms in several cell types, especially hepatocytes and macrophages. How these mechanisms function is not entirely clear, however.

HIF-signalling may also be involved in the link between OSA and NAFLD progression generally, and regarding inflammation in particular. Severity of nocturnal hypoxia in OSA correlates with NAFLD/NASH severity, including liver inflammation, independent of other risk factors in patients (25), and subjecting mice to CIH in order to mimic OSA leads to increased liver HIF1α, TNFα, and NF-κB (108). OSA induced inflammation may be mediated in part by changes in the balance between anti-inflammatory regulatory T cells and pro-inflammatory Th17 helper T cells (109). In mice fed a HFD, this ratio was shifted toward the pro-inflammatory Th17 cells, and this shift was even greater when CIH was superimposed through injection of sodium nitrite. Interference of HIF1α partially normalised this shift in the CIH and HFD exposed mice, and in hypoxic T-cells *in vitro*. This suggests HIF signalling in patients with NAFLD/NASH and OSA may induce or worsen inflammation, though more studies are needed to confirm this.

## Open Questions

The evidence currently available highlights potential mechanisms by which HIF signalling may be involved in several key aspects of NAFLD, namely steatosis, inflammation and fibrosis. Further work is required to confirm many of these mechanisms and provide a more detailed understanding, and to determine whether targeting HIF signalling is a viable treatment strategy to improve these aspects of the pathology. It also remains uncertain what drives liver hypoxia and HIF activation in NAFLD in the first place.

While high fat feeding has been shown to induce liver hypoxia even in a relatively short time frame (10), it has not yet been determined what processes lead to this. It also remains unclear whether ROS production plays a role in HIF induction in NAFLD. Short-term feeding of NAFLD inducing diets combined with measurement of oxidative metabolism [e.g., using mitochondrial respirometry (110) or metabolomics, especially with isotope tracing (111, 112)] in the liver could elucidate whether development of liver hypoxia is preceded by increased oxygen consumption. Concomitantly, measurement of ROS markers [such as thiol (113) or lipid peroxidation (114)] could show whether ROS production is likely to play a role in HIF activation, which could be followed up with *in vitro* studies using ROS scavengers to investigate whether this prevents HIF activation in *in vitro* models of NAFLD. Investigation of SIRT4 in this context could also be valuable as reduced levels of this sirtuin have been demonstrated in patients with NAFLD (19) and it has been proposed that this may be a driver of increased ROS production in this disease.

It has been demonstrated that HIF2α activation in normoxia can limit FAO in hepatocytes (32). However, as demonstrated by the observation that lipogenic gene expression is decreased in mice with HIF2α activation due to *Vhl* disruption (32), while it is increased in NAFLD rodent models (9) [which also show HIF2α activation (3)], this does not necessarily mean that HIF2α in NAFLD also reduces FAO. Studies that investigate the function and expression of enzymes involved in FAO, and of the key cellular organelle in oxidative metabolism, the mitochondrion, in NAFLD models (with HIF2α deletion or pharmacological inhibition) and patients would help elucidate this. More detailed metabolomic studies in these settings may also provide further insight into how HIF2α activation affects metabolism in NAFLD.

In many cases, details of the signalling pathways by which HIF activation contributes to NAFLD and NASH remain unclear. This includes the pathways leading to increased expression of lipogenic, fibrogenic, and pro-inflammatory genes. Biochemical and molecular biology techniques such as chromatin immunoprecipitation and co-immunoprecipitation may provide targets for further investigation. *In vitro* studies may prove useful to probe these targets due to the greater ease of deletion and overexpression of genes. However, the current lack of consistent *in vitro* NAFLD models may make this more challenging.

Currently, it is unclear to what extent OSA is required for HIF activation in patients with NAFLD, and whether HIF activation resulting from OSA differs in its effects on NAFLD from HIF activation without OSA. This is likely to be the case due to the hypoxia-reoxygenation cycles inherent to OSA, which may affect activation of HIFs (e.g., by favouring HIF1α over HIF2α activation) and other co-activated pathways. Further investigations into how closely linked HIF activation and OSA are in patients with NAFLD would be useful, and studies in animal models of NAFLD exposed to CIH—a way of mimicking OSA in rodents, which do not develop OSA spontaneously—could provide insight into whether and how these pathophysiological mechanisms differ.

The ultimate goal of understanding the involvement of HIF signalling in NAFLD would be to attempt to treat the disease by targeting this pathway. Current evidence supports the use of animal studies to investigate this, and both HIF1α and HIF2α antagonists have been developed, largely with a view to treating cancers (8). Early studies, looking for example at the effect of HIF2α antagonism in HFD induced hepatosteatosis in mice have shown promising results (9), but studies in more severe models of NAFLD and NASH do still need be conducted.

Finally, while this review has focussed on the role of HIF signalling in the liver, some studies point toward roles of HIFs in other tissues and organs that are likely to impact on NAFLD and outcomes in NAFLD. For example, HIF activation in adipocytes (115) and adipose tissue macrophages (116) has been shown to affect insulin resistance, which is likely to affect NAFLD development. Further, the close links between gut and liver are likely to be involved in NAFLD, as shown by the association between inflammatory bowel disease and NAFLD (117). HIFs are known to play an important role in inflammation in the intestine (118) and may be an important part of this inter-organ link. Indeed, HIF activation in the intestine can affect NAFLD directly (119). The role of HIFs in other organs in the broader context of metabolic disease has recently been reviewed elsewhere (120), and better understanding of this, and how it may affect interactions between other organs and the liver is likely to aid in the development of therapeutic strategies for NAFLD.

## Conclusion

In conclusion, considerable evidence points toward HIF activation occurring in NAFLD and NASH, and having widespread, predominantly harmful effects. Both HIF1α and HIF2α activation appear to worsen inflammation, though the mechanisms involved in this require further study. Further, evidence from studies of fibrosis shows important HIF mediated mechanisms, including control of profibrotic gene expression in hepatocytes and HSCs, regulation of HSC activation and HIF mediated pathological angiogenesis, though only control of profibrotic gene expression has been demonstrated to occur in animal models of NAFLD and NASH. Evidence also highlights a role for HIFs, in particular HIF2α, in driving steatosis. Studies of HIF activation under normoxic conditions suggest that HIF2α can inhibit FAO, while studies that interfere with HIF2α activation in NAFLD *via* oxygen therapy or antagonism suggest that HIF2α drives lipogenesis. These mechanisms could explain the protective effect that *Hif2*α deletion has against steatosis in NAFLD. While some beneficial effects of HIF activation have been noted, such as a potential role in improving insulin sensitivity, on balance, HIF activation appears to be harmful in NAFLD, and may therefore be a useful therapeutic target. Further research is required to fully elucidate the mechanisms by which HIF activation contributes to NAFLD and NASH, in particular the effect on FAO, the signalling pathways involved in regulating the expression of lipogenic, fibrogenic, and pro-inflammatory genes, and the link between HIF signalling and OSA in NAFLD and NASH.

## Author Contributions

All authors listed have made a substantial, direct and intellectual contribution to the work, and approved it for publication.

## Funding

LH was supported by a 4-year PhD studentship Program funded by the Wellcome Trust (Grant Number: 220033/Z/19/Z). AM was supported by the Research Councils UK (Grant Number: EP/E500552/1).

## Conflict of Interest

The authors declare that the research was conducted in the absence of any commercial or financial relationships that could be construed as a potential conflict of interest.

## Publisher's Note

All claims expressed in this article are solely those of the authors and do not necessarily represent those of their affiliated organizations, or those of the publisher, the editors and the reviewers. Any product that may be evaluated in this article, or claim that may be made by its manufacturer, is not guaranteed or endorsed by the publisher.

## Abbreviations

*Acox*: Acyl coenzyme A oxidase
*Acta*: α smooth muscle actin (gene)
α-SMA: α smooth muscle actin (protein)
ATP: Adenosine triphosphate
BDL: Bile duct ligation
CD36: Cluster of differentiation 36
CIH: Chronic intermittent hypoxia
*Col1a1*: Type 1 Collagen
*Cpt1*: Carnitine palmitoyltransferase 1
ECM: Extracellular matrix
*Epas1*: Endothelial PAS domain containing protein (gene)
FAO: Fatty acid oxidation
*Fas*: Fatty acid synthase
HFD: High fat diet
HIF: Hypoxia-inducible factor
HRGP: Histidine rich glycoprotein
HSC: Hepatic stellate cell
IL-1b: Interleukin 1b
*Irs2*: Insulin receptor substrate 2
*Lox*: Lysyl oxygenase
MCP1: Macrophage attractant protein 1
NAFLD: Non-alcoholic fatty liver disease
NASH: Non-alcoholic steatohepatitis
NF-κB: nuclear factor kappa-light-chain-enhancer of activated B cells
OSA: Obstructive sleep apnea
PAI-1: Plasminogen activator-inhibitor 1
PDGF: Platelet derived growth factor
*Pepck*: Phosphoenolpyruvate carboxykinase
*Pgc1a*: Peroxisome proliferator-activated receptor gamma coactivator 1α
*Phd*: Prolyl hydroxylase domain protein (gene)
PPAR: Peroxisome proliferator-activated receptor
ROS: Reactive oxygen species
*Scd1*: Stearoyl coenzyme A-desaturase 1
*Tnfa*: Tumour necrosis factor 1α
*Vegf*: Vascular endothelial growth factor
*Vhl*: Von Hippel-Lindau protein (gene).

## References

[1] Younossi ZM. Non-alcoholic fatty liver disease - a global public health perspective. J Hepatol. (2019) 70:531–44. 10.1016/j.jhep.2018.10.033 30414863

[2] Calzadilla Bertot L Adams LA. The natural course of non-alcoholic fatty liver disease. Int J Mol Sci. (2016) 17:774. 10.3390/ijms17050774 27213358 PMC4881593

[3] Morello E Sutti S Foglia B Novo E Cannito S Bocca C. Hypoxia-inducible factor 2alpha drives nonalcoholic fatty liver progression by triggering hepatocyte release of histidine-rich glycoprotein. Hepatology. (2018) 67:2196–214. 10.1002/hep.29754 29266399

[4] Duan C. Hypoxia-inducible factor 3 biology: complexities and emerging themes. Am J Physiol Cell Physiol. (2016) 310:C260–9. 10.1152/ajpcell.00315.2015 26561641

[5] Kaelin WG Jr. Ratcliffe PJ. Oxygen sensing by metazoans: the central role of the HIF hydroxylase pathway. Mol Cell. (2008) 30:393–402. 10.1016/j.molcel.2008.04.009 18498744

[6] Dengler VL Galbraith M Espinosa JM. Transcriptional regulation by hypoxia inducible factors. Crit Rev Biochem Mol Biol. (2014) 49:1–15. 10.3109/10409238.2013.838205 24099156 PMC4342852

[7] Lee JW Ko J Ju C Eltzschig HK. Hypoxia signaling in human diseases and therapeutic targets. Exp Mol Med. (2019) 51:1–13. 10.1038/s12276-019-0235-1 31221962 PMC6586801

[8] Yu T Tang B Sun X. Development of inhibitors targeting hypoxia-inducible factor 1 and 2 for cancer therapy. Yonsei Med J. (2017) 58:489–96. 10.3349/ymj.2017.58.3.489 28332352 PMC5368132

[9] Feng Z Zou X Chen Y Wang H Duan Y Bruick RK. Modulation of HIF-2alpha PAS-B domain contributes to physiological responses. Proc Natl Acad Sci USA. (2018) 115:13240–5. 10.1073/pnas.1810897115 30523118 PMC6310796

[10] Zhang X Huang C Li X Shangguan Z Wei W Liu S. HFD and HFD-provoked hepatic hypoxia act as reciprocal causation for NAFLD via HIF-independent signaling. BMC Gastroenterol. (2020) 20:366. 10.1186/s12876-020-01515-5 33143650 PMC7640429

[11] Kietzmann T. Liver zonation in health and disease: hypoxia and hypoxia-inducible transcription factors as concert masters. Int J Mol Sci. (2019) 20:2347. 10.3390/ijms20092347 31083568 PMC6540308

[12] Mantena SK Vaughn DP Andringa KK Eccleston HB King AL Abrams GA. High fat diet induces dysregulation of hepatic oxygen gradients and mitochondrial function *in vivo*. Biochem J. (2009) 417:183–93. 10.1042/BJ20080868 18752470 PMC2637578

[13] Aguilera KY Brekken RA. Hypoxia studies with pimonidazole *in vivo*. Bio Protoc. (2014) 4:e1254. 10.21769/BioProtoc.1254 27453908 PMC4956402

[14] Carabelli J Burgueno AL Rosselli MS Gianotti TF Lago NR Pirola CJ. High fat diet-induced liver steatosis promotes an increase in liver mitochondrial biogenesis in response to hypoxia. J Cell Mol Med. (2011) 15:1329–38. 10.1111/j.1582-4934.2010.01128.x 20629985 PMC4373333

[15] Simoes ICM Janikiewicz J Bauer J Karkucinska-Wieckowska A Kalinowski P Dobrzyn A. Fat and sugar-a dangerous duet. A comparative review on metabolic remodeling in rodent models of nonalcoholic fatty liver disease. Nutrients. (2019) 11. 10.3390/nu11122871 31771244 PMC6950566

[16] Movafagh S Crook S Vo K. Regulation of hypoxia-inducible factor-1a by reactive oxygen species: new developments in an old debate. J Cell Biochem. (2015) 116:696–703. 10.1002/jcb.25074 25546605

[17] Vial G Dubouchaud H Couturier K Cottet-Rousselle C Taleux N Athias A. Effects of a high-fat diet on energy metabolism and ROS production in rat liver. J Hepatol. (2011) 54:348–56. 10.1016/j.jhep.2010.06.044 21109325

[18] Anavi S Hahn-Obercyger M Madar Z Tirosh O. Mechanism for HIF-1 activation by cholesterol under normoxia: a redox signaling pathway for liver damage. Free Radic Biol Med. (2014) 71:61–9. 10.1016/j.freeradbiomed.2014.03.007 24632196

[19] Tarantino G Finelli C Scopacasa F Pasanisi F Contaldo F Capone D. Circulating levels of sirtuin 4, a potential marker of oxidative metabolism, related to coronary artery disease in obese patients suffering from NAFLD, with normal or slightly increased liver enzymes. Oxid Med Cell Longev. (2014) 2014:920676. 10.1155/2014/920676 25045415 PMC4086623

[20] Selak MA Armour SM MacKenzie ED Boulahbel H Watson DG Mansfield KD. Succinate links TCA cycle dysfunction to oncogenesis by inhibiting HIF-alpha prolyl hydroxylase. Cancer Cell. (2005) 7:77–85. 10.1016/j.ccr.2004.11.022 15652751

[21] Joo HY Yun M Jeong J Park ER Shin HJ Woo SR. SIRT1 deacetylates and stabilizes hypoxia-inducible factor-1alpha (HIF-1alpha) via direct interactions during hypoxia. Biochem Biophys Res Commun. (2015) 462:294–300. 10.1016/j.bbrc.2015.04.119 25979359

[22] Wu T Liu YH Fu YC Liu XM Zhou XH. Direct evidence of sirtuin downregulation in the liver of non-alcoholic fatty liver disease patients. Ann Clin Lab Sci. (2014) 44:410–8. 25361925

[23] Sforza E Roche F. Chronic intermittent hypoxia and obstructive sleep apnea: an experimental and clinical approach. Hypoxia. (2016) 4:99–108. 10.2147/HP.S103091 27800512 PMC5085272

[24] Romero-Corral A Caples SM Lopez-Jimenez F Somers VK. Interactions between obesity and obstructive sleep apnea: implications for treatment. Chest. (2010) 137:711–9. 10.1378/chest.09-0360 20202954 PMC3021364

[25] Aron-Wisnewsky J Minville C Tordjman J Levy P Bouillot JL Basdevant A. Chronic intermittent hypoxia is a major trigger for non-alcoholic fatty liver disease in morbid obese. J Hepatol. (2012) 56:225–33. 10.1016/j.jhep.2011.04.022 21703181

[26] Tarantino G Citro V Capone D. Nonalcoholic fatty liver disease: a challenge from mechanisms to therapy. J Clin Med. (2019) 9:15. 10.3390/jcm9010015 31861591 PMC7019297

[27] Hu CJ Wang LY Chodosh LA Keith B Simon MC. Differential roles of hypoxia-inducible factor 1alpha (HIF-1alpha) and HIF-2alpha in hypoxic gene regulation. Mol Cell Biol. (2003) 23:9361–74. 10.1128/MCB.23.24.9361-9374.2003 14645546 PMC309606

[28] Wu R Chang HC Khechaduri A Chawla K Tran M Chai X. Cardiac-specific ablation of ARNT leads to lipotoxicity and cardiomyopathy. J Clin Invest. (2014) 124:4795–806. 10.1172/JCI76737 25329697 PMC4347233

[29] Polotsky VY Patil SP Savransky V Laffan A Fonti S Frame LA. Obstructive sleep apnea, insulin resistance, and steatohepatitis in severe obesity. Am J Respir Crit Care Med. (2009) 179:228–34. 10.1164/rccm.200804-608OC 18990675 PMC2633055

[30] Ochiai D Goda N Hishiki T Kanai M Senoo-Matsuda N Soga T. Disruption of HIF-1alpha in hepatocytes impairs glucose metabolism in diet-induced obesity mice. Biochem Biophys Res Commun. (2011) 415:445–9. 10.1016/j.bbrc.2011.10.089 22051049 PMC6592821

[31] Bugianesi E Moscatiello S Ciaravella MF Marchesini G. Insulin resistance in nonalcoholic fatty liver disease. Curr Pharm Des. (2010) 16:1941–51. 10.2174/138161210791208875 20370677

[32] Rankin EB Rha J Selak MA Unger TL Keith B Liu Q. Hypoxia-inducible factor 2 regulates hepatic lipid metabolism. Mol Cell Biol. (2009) 29:4527–38. 10.1128/MCB.00200-09 19528226 PMC2725738

[33] Kucejova B Sunny NE Nguyen AD Hallac R Fu X Pena-Llopis S. Uncoupling hypoxia signaling from oxygen sensing in the liver results in hypoketotic hypoglycemic death. Oncogene. (2011) 30:2147–60. 10.1038/onc.2010.587 21217781 PMC3135264

[34] Qu A Taylor M Xue X Matsubara T Metzger D Chambon P. Hypoxia-inducible transcription factor 2alpha promotes steatohepatitis through augmenting lipid accumulation, inflammation, and fibrosis. Hepatology. (2011) 54:472–83. 10.1002/hep.24400 21538443 PMC3145012

[35] Grygiel-Gorniak B. Peroxisome proliferator-activated receptors and their ligands: nutritional and clinical implications–a review. Nutr J. (2014) 13:17. 10.1186/1475-2891-13-17 24524207 PMC3943808

[36] Mooli RGR Rodriguez J Takahashi S Solanki S Gonzalez FJ Ramakrishnan SK. Hypoxia via ERK signaling inhibits hepatic PPARalpha to promote fatty liver. Cell Mol Gastroenterol Hepatol. (2021) 12:585–97. 10.1016/j.jcmgh.2021.03.011 33798787 PMC8258975

[37] Cao R Zhao X Li S Zhou H Chen W Ren L. Hypoxia induces dysregulation of lipid metabolism in HepG2 cells via activation of HIF-2alpha. Cell Physiol Biochem. (2014) 34:1427–41. 10.1159/000366348 25323790

[38] Liu Y Ma Z Zhao C Wang Y Wu G Xiao J. HIF-1alpha and HIF-2alpha are critically involved in hypoxia-induced lipid accumulation in hepatocytes through reducing PGC-1alpha-mediated fatty acid beta-oxidation. Toxicol Lett. (2014) 226:117–23. 10.1016/j.toxlet.2014.01.033 24503013

[39] Chen J Chen J Fu H Li Y Wang L Luo S. Hypoxia exacerbates nonalcoholic fatty liver disease via the HIF-2alpha/PPARalpha pathway. Am J Physiol Endocrinol Metab. (2019) 317:E710–22. 10.1152/ajpendo.00052.2019 31430204

[40] Yu L Wang H Han X Liu H Zhu D Feng W. Oxygen therapy alleviates hepatic steatosis by inhibiting hypoxia-inducible factor-2alpha. J Endocrinol. (2020) 246:57–67. 10.1530/JOE-19-0555 32369776

[41] Arai T Tanaka M Goda N. HIF-1-dependent lipin1 induction prevents excessive lipid accumulation in choline-deficient diet-induced fatty liver. Sci Rep. (2018) 8:14230. 10.1038/s41598-018-32586-w 30242180 PMC6155071

[42] Donnelly KL Smith CI Schwarzenberg SJ Jessurun J Boldt MD Parks EJ. Sources of fatty acids stored in liver and secreted via lipoproteins in patients with nonalcoholic fatty liver disease. J Clin Invest. (2005) 115:1343–51. 10.1172/JCI23621 15864352 PMC1087172

[43] Lambert JE Ramos-Roman MA Browning JD Parks EJ. Increased *de novo* lipogenesis is a distinct characteristic of individuals with nonalcoholic fatty liver disease. Gastroenterology. (2014) 146:726–35. 10.1053/j.gastro.2013.11.049 24316260 PMC6276362

[44] Beysen C Schroeder P Wu E Brevard J Ribadeneira M Lu W. Inhibition of fatty acid synthase with FT-4101 safely reduces hepatic *de novo* lipogenesis and steatosis in obese subjects with non-alcoholic fatty liver disease: results from two early-phase randomized trials. Diabetes Obes Metab. (2021) 23:700–10. 10.1111/dom.14272 33289350 PMC7898808

[45] Rey E Melendez-Rodriguez F Maranon P Gil-Valle M Carrasco AG Torres-Capelli M. Hypoxia-inducible factor 2alpha drives hepatosteatosis through the fatty acid translocase CD36. Liver Int. (2020) 40:2553–67. 10.1111/liv.14519 32432822 PMC7539965

[46] Rey E Del Pozo-Maroto E Maranon P Beeler B Garcia-Garcia Y Landete P. Intrahepatic expression of fatty acid translocase CD36 is increased in obstructive sleep apnea. Front Med. (2020) 7:450. 10.3389/fmed.2020.00450 32850919 PMC7431763

[47] Li J Grigoryev DN Ye SQ Thorne L Schwartz AR Smith PL. Chronic intermittent hypoxia upregulates genes of lipid biosynthesis in obese mice. J Appl Physiol. (2005) 99:1643–8. 10.1152/japplphysiol.00522.2005 16037401

[48] Shin MK Drager LF Yao Q Bevans-Fonti S Yoo DY Jun JC. Metabolic consequences of high-fat diet are attenuated by suppression of HIF-1alpha. PLoS ONE. (2012) 7:e46562. 10.1371/journal.pone.0046562 23049707 PMC3462192

[49] Wei K Piecewicz SM McGinnis LM Taniguchi CM Wiegand SJ Anderson K. A liver Hif-2alpha-Irs2 pathway sensitizes hepatic insulin signaling and is modulated by Vegf inhibition. Nat Med. (2013) 19:1331–7. 10.1038/nm.3295 24037094 PMC3795838

[50] Taniguchi CM Finger EC Krieg AJ Wu C Diep AN LaGory EL. Cross-talk between hypoxia and insulin signaling through Phd3 regulates hepatic glucose and lipid metabolism and ameliorates diabetes. Nat Med. (2013) 19:1325–30. 10.1038/nm.3294 24037093 PMC4089950

[51] Rinella ME Sanyal AJ. Management of NAFLD. a stage-based approach. Nat Rev Gastroenterol Hepatol. (2016) 13:196–205. 10.1038/nrgastro.2016.3 26907882

[52] Singh S Allen AM Wang Z Prokop LJ Murad MH Loomba R. Fibrosis progression in nonalcoholic fatty liver vs nonalcoholic steatohepatitis: a systematic review and meta-analysis of paired-biopsy studies. Clin Gastroenterol Hepatol. (2015) 13:643–54 e1–9; quiz e39–40. 10.1016/j.cgh.2014.04.014 24768810 PMC4208976

[53] Kim D Kim WR Kim HJ Therneau TM. Association between noninvasive fibrosis markers and mortality among adults with nonalcoholic fatty liver disease in the United States. Hepatology. (2013) 57:1357–65. 10.1002/hep.26156 23175136 PMC3622816

[54] Ekstedt M Hagstrom H Nasr P Fredrikson M Stal P Kechagias S. Fibrosis stage is the strongest predictor for disease-specific mortality in NAFLD after up to 33 years of follow-up. Hepatology. (2015) 61:1547–54. 10.1002/hep.27368 25125077

[55] Trak-Smayra V Paradis V Massart J Nasser S Jebara V Fromenty B. Pathology of the liver in obese and diabetic ob/ob and db/db mice fed a standard or high-calorie diet. Int J Exp Pathol. (2011) 92:413–21. 10.1111/j.1365-2613.2011.00793.x 22118645 PMC3248077

[56] Mesarwi OA Shin MK Bevans-Fonti S Schlesinger C Shaw J Polotsky VY. Hepatocyte hypoxia inducible factor-1 mediates the development of liver fibrosis in a mouse model of nonalcoholic fatty liver disease. PLoS ONE. (2016) 11:e0168572. 10.1371/journal.pone.0168572 28030556 PMC5193414

[57] Boland ML Oro D Tolbol KS Thrane ST Nielsen JC Cohen TS. Towards a standard diet-induced and biopsy-confirmed mouse model of non-alcoholic steatohepatitis: impact of dietary fat source. World J Gastroenterol. (2019) 25:4904–20. 10.3748/wjg.v25.i33.4904 31543682 PMC6737317

[58] Rinella ME Green RM. The methionine-choline deficient dietary model of steatohepatitis does not exhibit insulin resistance. J Hepatol. (2004) 40:47–51. 10.1016/j.jhep.2003.09.020 14672613

[59] Van Campenhout S Van Vlierberghe H Devisscher L. Common bile duct ligation as model for secondary biliary cirrhosis. Methods Mol Biol. (2019) 1981:237–47. 10.1007/978-1-4939-9420-5_15 31016658

[60] Son G Iimuro Y Seki E Hirano T Kaneda Y Fujimoto J. Selective inactivation of NF-kappaB in the liver using NF-kappaB decoy suppresses CCl4-induced liver injury and fibrosis. Am J Physiol Gastrointest Liver Physiol. (2007) 293:G631–9. 10.1152/ajpgi.00185.2007 17640975

[61] Dong S Chen QL Song YN Sun Y Wei B Li XY. Mechanisms of CCl4-induced liver fibrosis with combined transcriptomic and proteomic analysis. J Toxicol Sci. (2016) 41:561–72. 10.2131/jts.41.561 27452039

[62] Scholten D Trebicka J Liedtke C Weiskirchen R. The carbon tetrachloride model in mice. Lab Anim. (2015) 49:4–11. 10.1177/0023677215571192 25835733

[63] Tolba R Kraus T Liedtke C Schwarz M Weiskirchen R. Diethylnitrosamine (DEN)-induced carcinogenic liver injury in mice. Lab Anim. (2015) 49:59–69. 10.1177/0023677215570086 25835739

[64] Mesarwi OA Shin MK Drager LF Bevans-Fonti S Jun JC Putcha N. Lysyl oxidase as a serum biomarker of liver fibrosis in patients with severe obesity and obstructive sleep apnea. Sleep. (2015) 38:1583–91. 10.5665/sleep.5052 26085300 PMC4576332

[65] Pavlacky J Polak J. Technical feasibility and physiological relevance of hypoxic cell culture models. Front Endocrinol. (2020) 11:57. 10.3389/fendo.2020.00057 32153502 PMC7046623

[66] Hernandez A Reyes D Geng Y Arab JP Cabrera D Sepulveda R. Extracellular vesicles derived from fat-laden hepatocytes undergoing chemical hypoxia promote a pro-fibrotic phenotype in hepatic stellate cells. Biochim Biophys Acta Mol Basis Dis. (2020) 1866:165857. 10.1016/j.bbadis.2020.165857 32512191

[67] Jusman SW Halim A Wanandi SI Sadikin M. Expression of hypoxia-inducible factor-1alpha (HIF-1alpha) related to oxidative stress in liver of rat-induced by systemic chronic normobaric hypoxia. Acta Med Indones. (2010) 42:17–23. 20305327

[68] Dukhande VV Sharma GC Lai JC Farahani R. Chronic hypoxia-induced alterations of key enzymes of glucose oxidative metabolism in developing mouse liver are mTOR dependent. Mol Cell Biochem. (2011) 357:189–97. 10.1007/s11010-011-0889-z 21625955

[69] Chopra S Polotsky VY Jun JC. Sleep apnea research in animals. Past, present, and future. Am J Respir Cell Mol Biol. (2016) 54:299–305. 10.1165/rcmb.2015-0218TR 26448201 PMC4821036

[70] Wu W Li W Wei J Wang C Yao Y Zhu W. Chronic intermittent hypoxia accelerates liver fibrosis in rats with combined hypoxia and nonalcoholic steatohepatitis via angiogenesis rather than endoplasmic reticulum stress. Acta Biochim Biophys Sin. (2019) 51:159–67. 10.1093/abbs/gmy169 30668625

[71] Takayama F Egashira T Kawasaki H Mankura M Nakamoto K Okada S. A novel animal model of nonalcoholic steatohepatitis (NASH): hypoxemia enhances the development of NASH. J Clin Biochem Nutr. (2009) 45:335–40. 10.3164/jcbn.09-29 19902025 PMC2771256

[72] Corpechot C Barbu V Wendum D Kinnman N Rey C Poupon R. Hypoxia-induced VEGF and collagen I expressions are associated with angiogenesis and fibrogenesis in experimental cirrhosis. Hepatology. (2002) 35:1010–21. 10.1053/jhep.2002.32524 11981751

[73] Tugues S Fernandez-Varo G Munoz-Luque J Ros J Arroyo V Rodes J. Antiangiogenic treatment with sunitinib ameliorates inflammatory infiltrate, fibrosis, and portal pressure in cirrhotic rats. Hepatology. (2007) 46:1919–26. 10.1002/hep.21921 17935226

[74] Moon JO Welch TP Gonzalez FJ Copple BL. Reduced liver fibrosis in hypoxia-inducible factor-1alpha-deficient mice. Am J Physiol Gastrointest Liver Physiol. (2009) 296:G582–92. 10.1152/ajpgi.90368.2008 19136383 PMC2660171

[75] Moczydlowska J Miltyk W Hermanowicz A Lebensztejn DM Palka JA Debek W. HIF-1 alpha as a key factor in bile duct ligation-induced liver fibrosis in rats. J Invest Surg. (2017) 30:41–6. 10.1080/08941939.2016.1183734 27260943

[76] Wang J Lu Z Xu Z Tian P Miao H Pan S. Reduction of hepatic fibrosis by overexpression of von Hippel-Lindau protein in experimental models of chronic liver disease. Sci Rep. (2017) 7:41038. 10.1038/srep41038 28112200 PMC5253623

[77] Han J He Y Zhao H Xu X. Hypoxia inducible factor-1 promotes liver fibrosis in nonalcoholic fatty liver disease by activating PTEN/p65 signaling pathway. J Cell Biochem. (2019) 120:14735–44. 10.1002/jcb.28734 31009107

[78] Bocca C Novo E Miglietta A Parola M. Angiogenesis and fibrogenesis in chronic liver diseases. Cell Mol Gastroenterol Hepatol. (2015) 1:477–88. 10.1016/j.jcmgh.2015.06.011 28210697 PMC5301407

[79] Copple BL Bustamante JJ Welch TP Kim ND Moon JO. Hypoxia-inducible factor-dependent production of profibrotic mediators by hypoxic hepatocytes. Liver Int. (2009) 29:1010–21. 10.1111/j.1478-3231.2009.02015.x 19302442 PMC3111079

[80] Ghosh AK Vaughan DE. PAI-1 in tissue fibrosis. J Cell Physiol. (2012) 227:493–507. 10.1002/jcp.22783 21465481 PMC3204398

[81] Liu J Li Y Liu L Wang Z Shi C Cheng Z. Double knockdown of PHD1 and Keap1 attenuated hypoxia-induced injuries in hepatocytes. Front Physiol. (2017) 8:291. 10.3389/fphys.2017.00291 28539891 PMC5423937

[82] Iwaisako K Brenner DA Kisseleva T. What's new in liver fibrosis? The origin of myofibroblasts in liver fibrosis. J Gastroenterol Hepatol. (2012) 27(Suppl 2):65–8. 10.1111/j.1440-1746.2011.07002.x 22320919 PMC4841268

[83] Copple BL Bai S Burgoon LD Moon JO. Hypoxia-inducible factor-1alpha regulates the expression of genes in hypoxic hepatic stellate cells important for collagen deposition and angiogenesis. Liver Int. (2011) 31:230–44. 10.1111/j.1478-3231.2010.02347.x 20880076 PMC3099214

[84] Copple BL Bai S Moon JO. Hypoxia-inducible factor-dependent production of profibrotic mediators by hypoxic Kupffer cells. Hepatol Res. (2010) 40:530–9. 10.1111/j.1872-034X.2010.00635.x 20412331 PMC2886188

[85] Copple BL Kaska S Wentling C. Hypoxia-inducible factor activation in myeloid cells contributes to the development of liver fibrosis in cholestatic mice. J Pharmacol Exp Ther. (2012) 341:307–16. 10.1124/jpet.111.189340 22271822 PMC3336817

[86] Schadde E Tsatsaris C Swiderska-Syn M Breitenstein S Urner M Schimmer R. Hypoxia of the growing liver accelerates regeneration. Surgery. (2017) 161:666–79. 10.1016/j.surg.2016.05.018 27436690

[87] Mejias M Garcia-Pras E Tiani C Miquel R Bosch J Fernandez M. Beneficial effects of sorafenib on splanchnic, intrahepatic, and portocollateral circulations in portal hypertensive and cirrhotic rats. Hepatology. (2009) 49:1245–56. 10.1002/hep.22758 19137587

[88] Yang L Kwon J Popov Y Gajdos GB Ordog T Brekken RA. Vascular endothelial growth factor promotes fibrosis resolution and repair in mice. Gastroenterology. (2014) 146:1339–50 e1. 10.1053/j.gastro.2014.01.061 24503129 PMC4001704

[89] Novo E Cannito S Zamara E Valfre di Bonzo L Caligiuri A Cravanzola C. Proangiogenic cytokines as hypoxia-dependent factors stimulating migration of human hepatic stellate cells. Am J Pathol. (2007) 170:1942–53. 10.2353/ajpath.2007.060887 17525262 PMC1899450

[90] Wang YQ Luk JM Ikeda K Man K Chu AC Kaneda K. Regulatory role of vHL/HIF-1alpha in hypoxia-induced VEGF production in hepatic stellate cells. Biochem Biophys Res Commun. (2004) 317:358–62. 10.1016/j.bbrc.2004.03.050 15063765

[91] Luedde T Schwabe RF. NF-kappaB in the liver–linking injury, fibrosis and hepatocellular carcinoma. Nat Rev Gastroenterol Hepatol. (2011) 8:108–18. 10.1038/nrgastro.2010.213 21293511 PMC3295539

[92] Oakley F Teoh V Ching ASG Bataller R Colmenero J Jonsson JR. Angiotensin II activates I kappaB kinase phosphorylation of RelA at Ser 536 to promote myofibroblast survival and liver fibrosis. Gastroenterology. (2009) 136:2334–44 e1. 10.1053/j.gastro.2009.02.081 19303015

[93] Bracken CP Whitelaw ML Peet DJ. Activity of hypoxia-inducible factor 2alpha is regulated by association with the NF-kappaB essential modulator. J Biol Chem. (2005) 280:14240–51. 10.1074/jbc.M409987200 15653678

[94] Rius J Guma M Schachtrup C Akassoglou K Zinkernagel AS Nizet V. NF-kappaB links innate immunity to the hypoxic response through transcriptional regulation of HIF-1alpha. Nature. (2008) 453:807–11. 10.1038/nature06905 18432192 PMC2669289

[95] D'Ignazio L Bandarra D Rocha S. NF-kappaB and HIF crosstalk in immune responses. FEBS J. (2016) 283:413–24. 10.1111/febs.13578 26513405 PMC4864946

[96] Cai H Bai Z Ge RL. Hypoxia-inducible factor-2 promotes liver fibrosis in non-alcoholic steatohepatitis liver disease via the NF-kappaB signalling pathway. Biochem Biophys Res Commun. (2021) 540:67–74. 10.1016/j.bbrc.2021.01.002 33450482

[97] Mesarwi OA Moya EA Zhen X Gautane M Zhao H Wegbrans Giro P. Hepatocyte HIF-1 and intermittent hypoxia independently impact liver fibrosis in murine NAFLD. Am J Respir Cell Mol Biol. (2021). 10.1165/rcmb.2020-0492OC 34003729 PMC8525205

[98] Yoshiji H Kuriyama S Yoshii J Ikenaka Y Noguchi R Hicklin DJ. Vascular endothelial growth factor and receptor interaction is a prerequisite for murine hepatic fibrogenesis. Gut. (2003) 52:1347–54. 10.1136/gut.52.9.1347 12912869 PMC1773781

[99] Palazon A Goldrath AW Nizet V Johnson RS. HIF transcription factors, inflammation, and immunity. Immunity. (2014) 41:518–28. 10.1016/j.immuni.2014.09.008 25367569 PMC4346319

[100] Cummins EP Keogh CE Crean D Taylor CT. The role of HIF in immunity and inflammation. Mol Aspects Med. (2016) 47-48:24–34. 10.1016/j.mam.2015.12.004 26768963

[101] Sadiku P Walmsley SR. Hypoxia and the regulation of myeloid cell metabolic imprinting: consequences for the inflammatory response. EMBO Rep. (2019) 20. 10.15252/embr.201847388 30872317 PMC6500960

[102] Watts ER Walmsley SR. Inflammation and hypoxia: HIF and PHD isoform selectivity. Trends Mol Med. (2019) 25:33–46. 10.1016/j.molmed.2018.10.006 30442494

[103] Taylor CT Cummins EP. The role of NF-kappaB in hypoxia-induced gene expression. Ann N Y Acad Sci. (2009) 1177:178–84. 10.1111/j.1749-6632.2009.05024.x 19845620

[104] Caligiuri A Gentilini A Marra F. Molecular pathogenesis of NASH. Int J Mol Sci. (2016) 17:1575. 10.3390/ijms17091575 27657051 PMC5037841

[105] Ouyang X Han SN Zhang JY Dioletis E Nemeth BT Pacher P. Digoxin suppresses pyruvate kinase M2-promoted HIF-1alpha transactivation in steatohepatitis. Cell Metab. (2018) 27:1156. 10.1016/j.cmet.2018.04.007 29719229

[106] Wang X de Carvalho Ribeiro M Iracheta-Vellve A Lowe P Ambade A Satishchandran A. Macrophage-specific hypoxia-inducible factor-1alpha contributes to impaired autophagic flux in nonalcoholic steatohepatitis. Hepatology. (2019) 69:545–63. 10.1002/hep.30215 30102772 PMC6351177

[107] Bartneck M Fech V Ehling J Govaere O Warzecha KT Hittatiya K. Histidine-rich glycoprotein promotes macrophage activation and inflammation in chronic liver disease. Hepatology. (2016) 63:1310–24. 10.1002/hep.28418 26699087

[108] da Rosa DP Forgiarini LF Baronio D Feijo CA Martinez D Marroni NP. Simulating sleep apnea by exposure to intermittent hypoxia induces inflammation in the lung and liver. Mediators Inflamm. (2012) 2012:879419. 10.1155/2012/879419 23226929 PMC3513737

[109] Liu J Li W Zhu W He W Zhao H Xiang Y. Chronic intermittent hypoxia promotes the development of experimental non-alcoholic steatohepatitis by modulating Treg/Th17 differentiation. Acta Biochim Biophys Sin. (2018) 50:1200–10. 10.1093/abbs/gmy131 30379980

[110] Ost M Doerrier C Gama-Perez P Moreno-Gomez S. Analysis of mitochondrial respiratory function in tissue biopsies and blood cells. Curr Opin Clin Nutr Metab Care. (2018) 21:336–42. 10.1097/MCO.0000000000000486 29939971

[111] Perakakis N Stefanakis K Mantzoros CS. The role of omics in the pathophysiology, diagnosis and treatment of non-alcoholic fatty liver disease. Metabolism. (2020) 111S:154320. 10.1016/j.metabol.2020.154320 32712221 PMC7377759

[112] Puchalska P Crawford PA. Application of stable isotope labels for metabolomics in studies in fatty liver disease. Methods Mol Biol. (2019) 1996:259–72. 10.1007/978-1-4939-9488-5_20 31127561

[113] Sutton TR Minnion M Barbarino F Koster G Fernandez BO Cumpstey AF. A robust and versatile mass spectrometry platform for comprehensive assessment of the thiol redox metabolome. Redox Biol. (2018) 16:359–80. 10.1016/j.redox.2018.02.012 29627744 PMC5953223

[114] Aguilar Diaz De Leon J Borges CR. Evaluation of oxidative stress in biological samples using the thiobarbituric acid reactive substances assay. J Vis Exp. (2020) 159:e61122. 10.3791/61122 32478759 PMC9617585

[115] Lee YS Kim JW Osborne O Oh DY Sasik R Schenk S. Increased adipocyte O_2_ consumption triggers HIF-1alpha, causing inflammation and insulin resistance in obesity. Cell. (2014) 157:1339–52. 10.1016/j.cell.2014.05.012 24906151 PMC4114226

[116] Choe SS Shin KC Ka S Lee YK Chun JS Kim JB. Macrophage HIF-2alpha ameliorates adipose tissue inflammation and insulin resistance in obesity. Diabetes. (2014) 63:3359–71. 10.2337/db13-1965 24947359

[117] Magri S Paduano D Chicco F Cingolani A Farris C Delogu G. Nonalcoholic fatty liver disease in patients with inflammatory bowel disease: beyond the natural history. World J Gastroenterol. (2019) 25:5676–86. 10.3748/wjg.v25.i37.5676 31602167 PMC6785525

[118] Shah YM. The role of hypoxia in intestinal inflammation. Mol Cell Pediatr. (2016) 3:1. 10.1186/s40348-016-0030-1 26812949 PMC4728161

[119] Xie C Yagai T Luo Y Liang X Chen T Wang Q. Activation of intestinal hypoxia-inducible factor 2alpha during obesity contributes to hepatic steatosis. Nat Med. (2017) 23:1298–308. 10.1038/nm.4412 29035368 PMC6410352

[120] Gonzalez FJ Xie C Jiang C. The role of hypoxia-inducible factors in metabolic diseases. Nat Rev Endocrinol. (2018) 15:21–32. 10.1038/s41574-018-0096-z 30275460 PMC6624429

